# Transcriptome Analysis of Viable but Non-Culturable *Brettanomyces bruxellensis* Induced by Hop Bitter Acids

**DOI:** 10.3389/fmicb.2022.902110

**Published:** 2022-05-30

**Authors:** Yang He, Junfeng Zhao, Hua Yin, Yuan Deng

**Affiliations:** ^1^State Key Laboratory of Biological Fermentation Engineering of Beer, Tsingtao Brewery Co. Ltd., Qingdao, China; ^2^College of Food Science and Engineering, Henan University of Science and Technology, Luoyang, China; ^3^Animal Products Processing Laboratory, Hunan Institute of Animal Husbandry and Veterinary Science, Changsha, China

**Keywords:** beer-spoilage yeast, *Brettanomyces bruxellensis*, viable but non-culturable state (VBNC), hop bitter acids, transcriptome

## Abstract

The viable but non-culturable (VBNC) state has been studied in detail in bacteria. However, it has received much less attention in eukaryotic cells. The induction of a VBNC beer-spoilage yeast (*Brettanomyces bruxellensis*) by hop bitter acids with different concentrations and its recovery were studied in this work. *B. bruxellensis* cells were completely induced into the VBNC state by treatment of 250 mg/L hop bitter acids for 2 h. The addition of catalase at a concentration of 2,000 U/plate on YPD agars enabled these VBNC cells to recover their culturability within 2 days. Moreover, the transcriptome profiling revealed that 267 and 197 genes were significantly changed upon VBNC state entry and resuscitation, respectively. The differentially expressed genes involved in the peroxisome activities, ABC transporter, organic acid metabolism, and TCA cycle were mainly downregulated in the VBNC cells. In contrast, the amino acid and carbohydrate metabolism, cell division, and DNA replication were promoted. This study supplies a theoretical basis for microbial risk assessment in the brewing industry.

## Introduction

Hop (*Humulus lupulus* L.) is an essential raw material for beer brewing, principally on account of the highly desirable bitterness, aroma, and antibacterial capability (Dresel et al., 2016). Hop bitter acids with poor water solubility are the most important components in hops (Behr et al., 2007; Behr and Vogel, 2010), which are isomerized to more water-soluble isohumulones as a result of high temperature in the wort boiling process (Hazelwood et al., 2010). Recently, it has been confirmed that isomerized bitter acids exhibit strong antimicrobial activity, especially against S*treptococcus* spp., *Lactobacillus* spp., and *Listeria monocytogenes* (Behr et al., 2007; Dresel et al., 2016). Hazelwood et al. (2010) demonstrated that hop bitter acids at an approximate concentration of 500 mg/L also could cause the yeast *Saccharomyces cerevisiae* growth inhibition, which was much higher than the concentrations inhibiting bacterial growth. *Brettanomyces bruxellensis* is a common beer-spoilage yeast due to its ability to produce off-flavors and high levels of acetic acid (Turvey et al., 2016). Therefore, it presents a major risk for the brewing industry and can lead to cost-intensive recall of contaminated products and damage to brand reputation. On the other hand, it is considered beneficial especially in Belgian sour beers and some novel beer styles in which *Brettanomyces* is even encouraged to use in secondary fermentation.

It is generally known that microorganisms are endowed with defense strategies to combat unfavorable environmental conditions, such as nutrient starvation, extreme temperatures and pH, and oxidative stress, as well as chemical toxicity (Wong and Wang, 2004; Deng et al., 2014, 2015; Zhao et al., 2017). The viable but non-culturable (VBNC) state is a main adaptive response of non-spore-forming bacteria against adverse external stresses (Ayrapetyan and Oliver, 2016; Deng et al., 2016; Piao et al., 2019; Wang et al., 2020). Until now, studies on the VBNC state have been focused mainly on pathogenic and spoilage bacteria (Zhao et al., 2017; Dong et al., 2020). VBNC cells fail to develop into colonies on routine culturing media, but they are alive and can regain their culturability under desirable conditions (Zhao et al., 2016; Liao et al., 2021). Although at least 90 bacterial species can enter the VBNC state, reports on the VBNC behavior in yeast are extremely scarce (Serpaggi et al., 2012; Salma et al., 2013; Liu et al., 2016, 2018; Xiao et al., 2022).

This study investigated the entry into the VBNC state of a beer spoilage yeast *B. bruxellensis* B36, subjected to various concentrations of hop bitter acids. The transcriptome analysis was further performed to understand the mechanisms underlying the formation of hop bitter acids-induced VBNC *B. bruxellensis*.

## Materials and Methods

### Yeast, Cultivation, and Chemicals

The beer spoilage *B. bruxellensis* B36 (CGMCC accession no. 12804; China General Microbiology Culture Collection Center) was previously isolated from contaminated ale beer and subsequently preserved in our laboratory. PCR assay was performed by amplified targeting the 18S rRNA gene for the identification of the *B. bruxellensis* B36 strain (Cai et al., 1996). The yeast strain was maintained on YPD agar (BD Difco Co. Ltd., Detroit, MI, USA). Wort production was conducted by using the mixture of 12-kg malt extract (Shanghai KingBee Biotechnology Co. Ltd., China) and 1 L of water, followed by boiling for 1 h and then cooling to room temperature. Isomerized hop bitter acids were obtained from Botanix Pharma. Ltd. (Tonbridge, Kent, UK), which consist of isohumulone: isocohumulone: isoadhumulone with ratio of 38: 49: 13.

### Formation of VBNC *B. bruxellensis* Cells

*B. bruxellensis* B36 was inoculated into a sterile 250 ml flask containing 100 ml of YPD broth, which was incubated at 30°C on a rotary shaker (120 rpm) to reach 7 log cells/ml. The *B. bruxellensis* cells were harvested by centrifugation at 10,000 × *g* for 10 min at 4°C after incubation, followed by inoculation in the prepared wort with hop bitter acids at final concentrations of 0, 150, 200, and 250 mg/L, respectively. Next, the resultant culture was shaken at 60 rpm for 10 h at 30°C. The cell suspensions were sampled every 2 h during the 12 h induction for the total, viable, and culturable cell counts. The induction experiments of VBNC *B. bruxellensis* were performed in triplicates.

### Cell Counting Procedures

Fluorescein diacetate (FDA) and propidium iodide (PI) nucleic acid staining dyes (Molecular Probes, Inc., Eugene, OR, USA) were used to examine the viable and dead populations as described in our previous study (Xiao et al., 2022). FDA can penetrate both viable and damaged cells, while PI only intercalates into the DNA of damaged cells. The stained cells were determined by using Guava easyCyte™ 8-8HT flow cytometer (Guava Technologies Inc., Hayward, CA, USA). The viable cells emitting green fluorescence and dead cells emitting red fluorescence were counted, respectively, and total yeast count is the sum of the numbers of viable cells and dead cells. In addition, the conventional YPD plate culture was carried out for counting of culturable cells as described elsewhere (Deng et al., 2018). The difference between the viable and culturable cell counts was identified as the number of VBNC cells.

### Recovery of VBNC *B. bruxellensis* Cells

The resuscitation experiments were conducted by the addition of different chemical agents into YPD agar plates according to our previous method (Piao et al., 2019). Briefly, 100 μl VBNC *B. bruxellensis* cells were plated onto YPD agar media with catalase, Tween 80, and Tween 20 (Sigma-Aldrich Co., St. Louis, MO, USA) at final concentrations of 2,000 U/plate, 0.15% v/v, and 0.15% v/v. Heat-denatured catalase (65°C for 30 min) was served as a control. Resuscitation from the VBNC state with changing medium concentration was also performed as follows. VBNC cells (100 μl) were plated on YPD agar with concentrations ranging from 10 to 200%, respectively. After incubation at 30°C for 14 days, the colonies were counted.

### Transcriptome Analysis

Total RNA was extracted from the *B. bruxellensis* cell samples using the TRIzol Reagent protocol (Invitrogen/Life Technologies, Carlsbad, CA, USA) according to the manufacturer's instructions. RNA concentration and integrity of each sample was measured using NanoDrop ND-1000 UV-VIS Spectrophotometer version 3.3.1 (Thermo Scientific, Wilmington, DE, USA). Ribosomal RNAs and trace DNA were removed by two rounds of treatment with the Ribo-Zero Kit (Epicenter Inc., Madison, WI, USA). Mixed with the fragmentation buffer, the mRNA was fragmented into short pieces and then reverse-transcribed into cDNA (target DNA). Sequencing libraries were constructed from 1 μg of total RNA as a template using the TruSeq RNA Sample Preparation Kit (Illumina, San Diego, CA, USA) and then sequenced using a paired-end protocol on an Illumina NovaSeq 6000 platform to produce over 150 bp of sequence reads.

The raw sequencing data were processed and filtered using Cutadapt v1.16 software to remove low-quality reads, and then mapped to the reference genome (GenBank accession no. ASM1107488v2) using Tophat 2 v2.0.14. The gene expression levels were quantified using RNA-seq by expectation-maximization (RSEM) to identify known genes. In order to evaluate both the reproducibility and accuracy, the average correlation coefficient among three replicas was calculated. When values were closer to 1, the reproducibility was better. The FPKM (Fragments per kb per million reads) method was further used to normalize the gene expression level and to eliminate the influence of different gene lengths and sequencing discrepancies. Differentially expressed genes (DEGs) across the different sample pairs were detected using the DESeq package v1.10.1. The *P*-value threshold was set using a false discovery rate <0.05 and the absolute value of log_2_ fold-change with FPKM > 1 to determine significant differences in gene expression. DEGs were then subjected to an enrichment analysis of Gene Ontology (GO) and Kyoto Encyclopedia of Genes and Genomes (KEGG) pathways.

### Statistical Analysis

All experiments were performed in triplicates. The Tukey's comparison test (Xlstat software) was used to perform ANOVA with the least square differences (LSD) method used for multiple comparisons of means (level of statistically significance at *P* < 0.05).

## Results and Discussion

### Formation and Recovery of VBNC *B. bruxellensis* Cells

**Figure 1:**
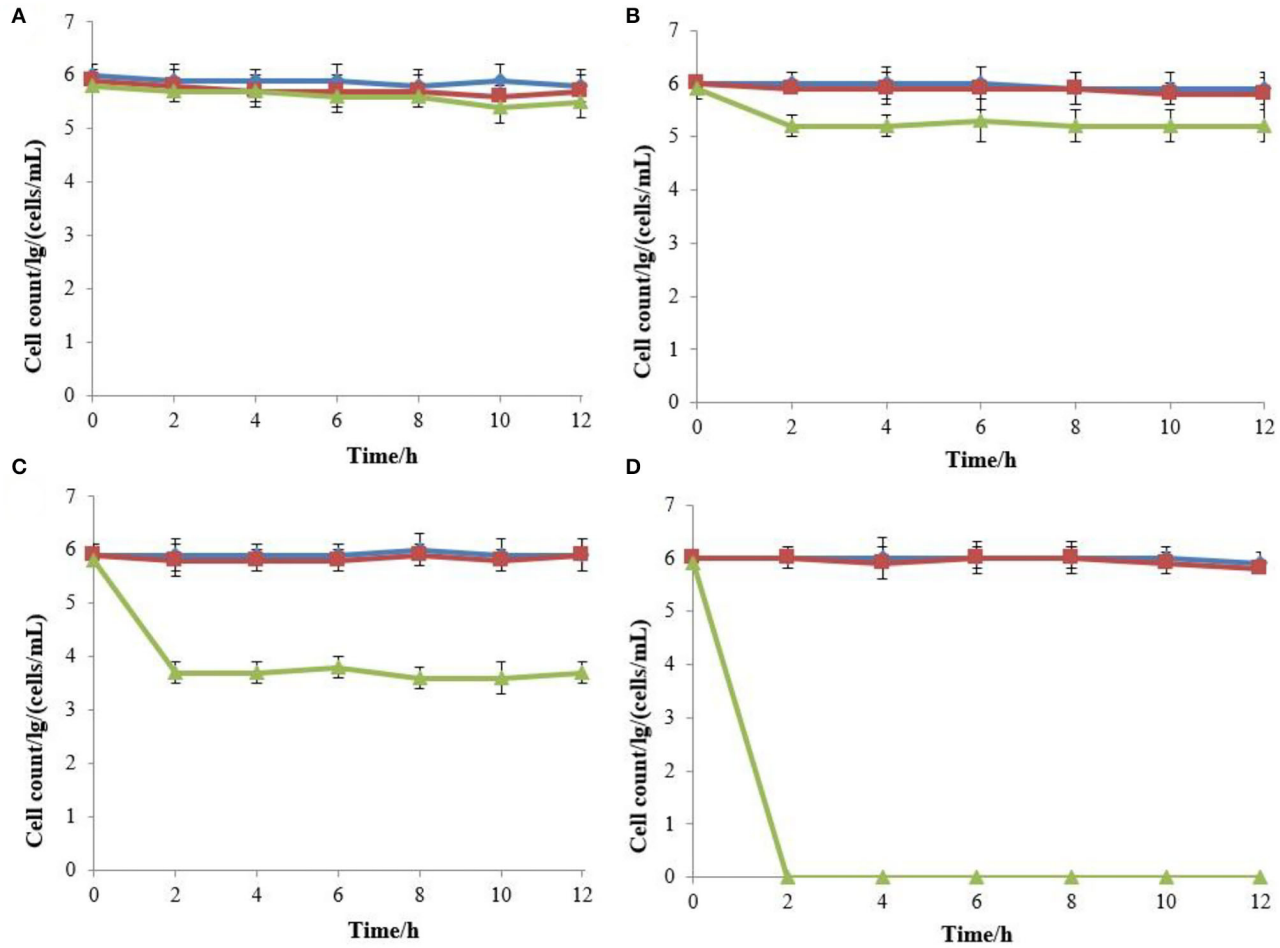
Total cell, viable cell, and culturable cell counts of *B. bruxellensis* B36 treated with various concentrations of isomerized hop extract [**(A)** 0 g/L; **(B)** 150 mg/L; **(C)** 200 mg/L; and **(D)** 250 mg/L]. Total cell counts (filled square); viable cell counts (filled triangle); culturable cell counts (filled circle).

**Figure 2:**
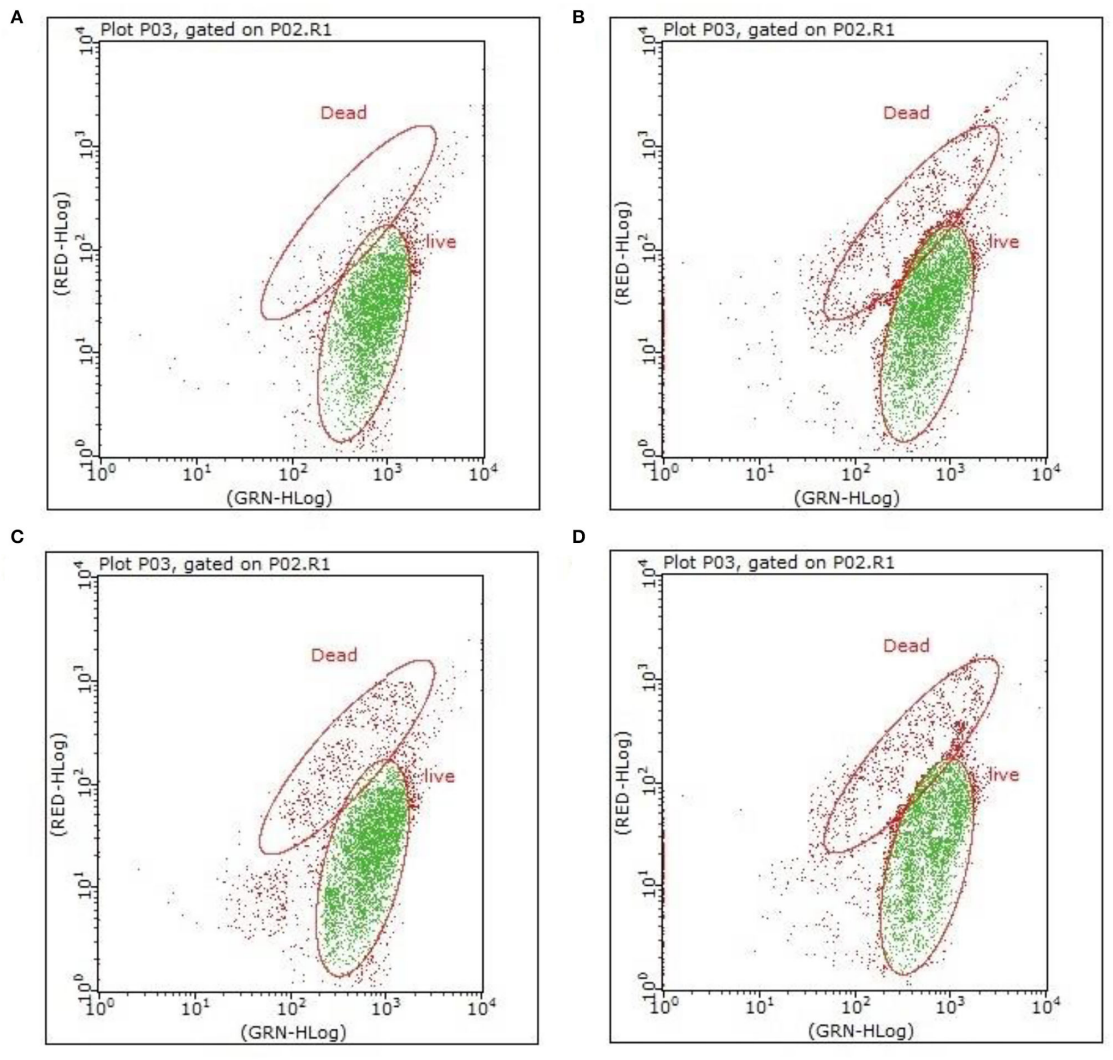
Flow cytometry analysis of VBNC *B. bruxellensis* cells induced by various concentrations of isomerized hop extract [**(A)** 0 g/L; **(B)** 150 mg/L; **(C)** 200 mg/L; and **(D)** 250 mg/L]. Both viable cells emitting green fluorescence and dead cells emitting red fluorescence are visualized simultaneously under appropriate conditions.

The effects of various concentrations of hop bitter acids on the viability and culturability of *B. bruxellensis* B36 is illustrated in Figure 1. Results obtained from flow cytometry showed that the number of total cells remained almost constant throughout the entire 12-day induction period after treatment with hop bitter acids ranging from 0 to 250 mg/L, while the number of viable cells decreased slightly (Figures 2A–D). Importantly, the culturable cell counts declined in varying degrees. *B. bruxellensis* cells lost culturability completely after treatment with 250 mg/L of hop bitter acids for 2 h. However, the number of viable cells simultaneously kept at a high level (Figures 2A–D), indicated that numerous *B. bruxellensis* cells were in the VBNC state. Additionally, the culturable cell counts treated with 150 and 200 mg/L of hop bitter acids for 2 h decreased gradually to 3 × 10^4^ and 7 × 10^2^ cells/mL, respectively, and then remained almost unchanged. The difference between the percentage of viable cells and culturable cells showed a part of the *B. bruxellensis* cells in the VBNC state. The VBNC *B. bruxellensis* was thus obtained by treatment with different concentrations of hop bitter acids. In addition, the *B. bruxellensis* cells in the VBNC state induced by the treatment with 250 mg/L of hop bitter acids could form 26 colonies on YPD agar with the addition of 2,000 U/plate of catalase after incubation at 30°C for 14 days. However, the recovery was ineffective when the VBNC cells were treated on YPD media without catalase and with heat-inactivated catalase or other chemicals (Table 1). Importantly, no colony appeared on the YPD agar plates upon changing medium concentration (data not shown). Hence, culturability of VBNC *B. bruxellensis* cells could be restored under the treatment of catalase.

**Table 1:** Effects of various supplements added in the YPD agar media on the recovery of *B. bruxellensis* B36.

**Supplement**	**Final concentration**	**Recovery time^**a**^**
CK	-	ND
Catalase	2,000 mg/L	2 h
Tween 80	0.15% v/v	ND
Tween 20	0.15% v/v	ND
Heat-denatured catalase	2,000 mg/L	ND

*CK, YPD agar plates without any supplementation*.

*ND, not detected*.

a *the day of the first observation of colonies*.

Previous studies have confirmed that the VBNC state can be successfully induced upon exposure to sulfur dioxide in *B. bruxellensis* and *S. cerevisiae* (Serpaggi et al., 2012; Salma et al., 2013; Capozzi et al., 2016). Sulfur dioxide is earlier identified as a chemical stressor for sterilization treatment in wine production (Du Toit et al., 2005). Divol and Lonvaud-Funel (2005) also found the presence of VBNC *Candida stellata* in wine. Furthermore, Bleve et al. (2003) indicated the occurrence of VBNC *B. bruxellensis* in other pasteurized foods. As far as we know, this present study is the first report on the existence of hop bitter acids-induced VBNC state *B. bruxellensis*. The hop bitter acids are hop-derived bitter components that consist of two related groups of compounds, the α- and β-acids. They are isomerized to more water-soluble matured hop bitter acids during wort boiling. These isomerized hop bitter acids are generally present at concentrations of 19.1–210 mg/L in beer (Fukuda et al., 2020). In view of our results, we propose that there may be some *B. bruxellensis* cells that can be hidden in beer by entering the VBNC state during the beer brewing and storage process due to the adaption to hop bitter acids. These VBNC *B. bruxellensis* cells also cause serious beer-spoilage incidents without being detected by conventional culture methods. However, since all experiments were conducted in YPD medium in the current work, the occurrence of VBNC *B. bruxellensis* cells under “natural conditions” in brewery environments should be further investigated in future studies.

Hop bitter acids can affect the cellular metal homeostasis by acting as strong iron and zinc chelators (Hazelwood et al., 2010). Metal iron deficiency or excess in yeast cells can induce the increase in free radical levels under certain circumstances resulting in cellular oxidative damage. Catalase as an important antioxidant and protective enzyme, is able to scavenge free radicals. Our results demonstrated that adding catalase to the media is an effective solution for restoring the culturability of VBNC *B. bruxellensis* due to the protection from oxidative stress in this study, which are consistent with the earlier findings of Wang et al. (2020) and Zhong et al. (2009) concerning the recovery of various bacterial species from the VBNC state. The antimicrobial activity of hop bitter acids are reported to be involved in the efficient transmembrane redox reaction causing cellular oxidative damage (Behr and Vogel, 2010). The VBNC *B. bruxellensis* could be stressed and sensitized to detoxify superoxide during phenotypic adaptation to hop bitter acids. Therefore, the antioxidant capacity of catalase might alleviate the stressor.

### Transcriptome Analysis

**Figure 3:**
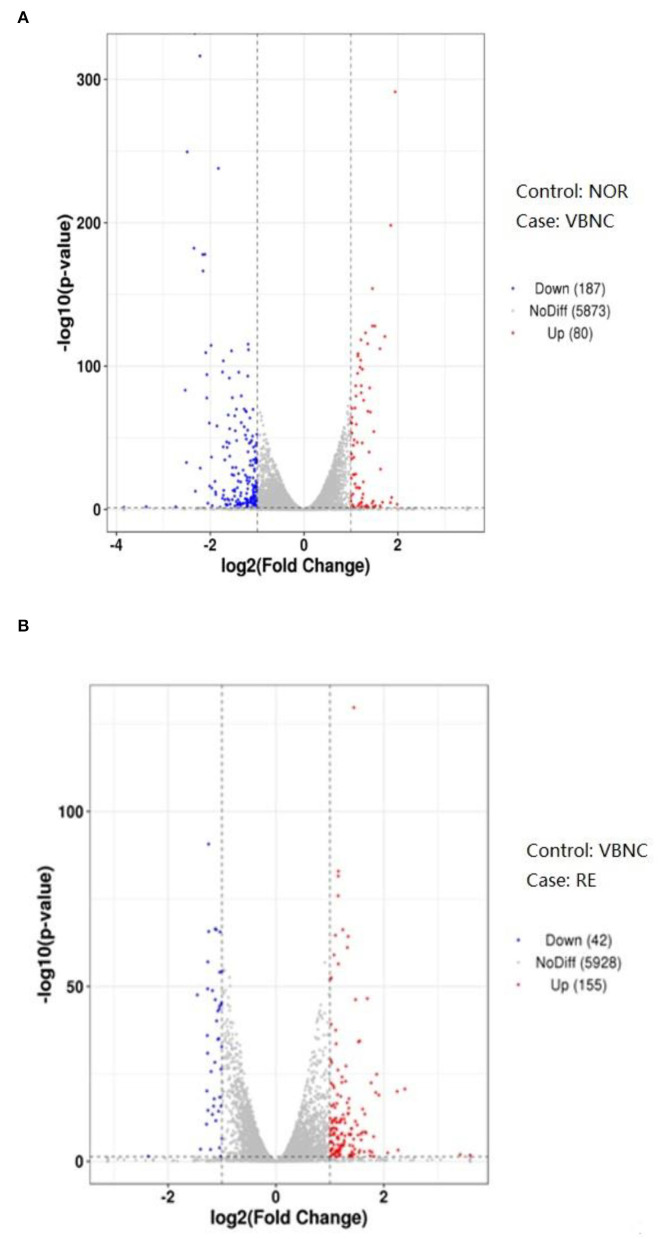
Volcano plots of differentially expressed genes between the normal and VBNC cells **(A)** and between the VBNC and resuscitated cells **(B)**.

Transcriptome analysis was performed in normal cells (NOR), VBNC cells (VBNC), and cells resuscitated after catalase treatment (RE) of *B. bruxellensis*. The differential gene expression of pairwise comparison between groups including NOR vs. VBNC, and VBNC vs. RE, are presented by volcano plots in Figure 3. The vertical and horizontal coordinates in the volcano plot denote respectively the negative log10 (*P*-value) and log2 (fold-change) of the two group samples; the red (up-regulated) and blue (down-regulated) spots reveal remarkable gene expression changes. A total of 267 DEGs were identified after the formation of VBNC state (NOR vs. VBNC), of which 80 were up-regulated and 187 down-regulated. Moreover, 197 DEGs were obtained by comparison of the gene expression levels between VBNC and RE.

**Figure 4:**
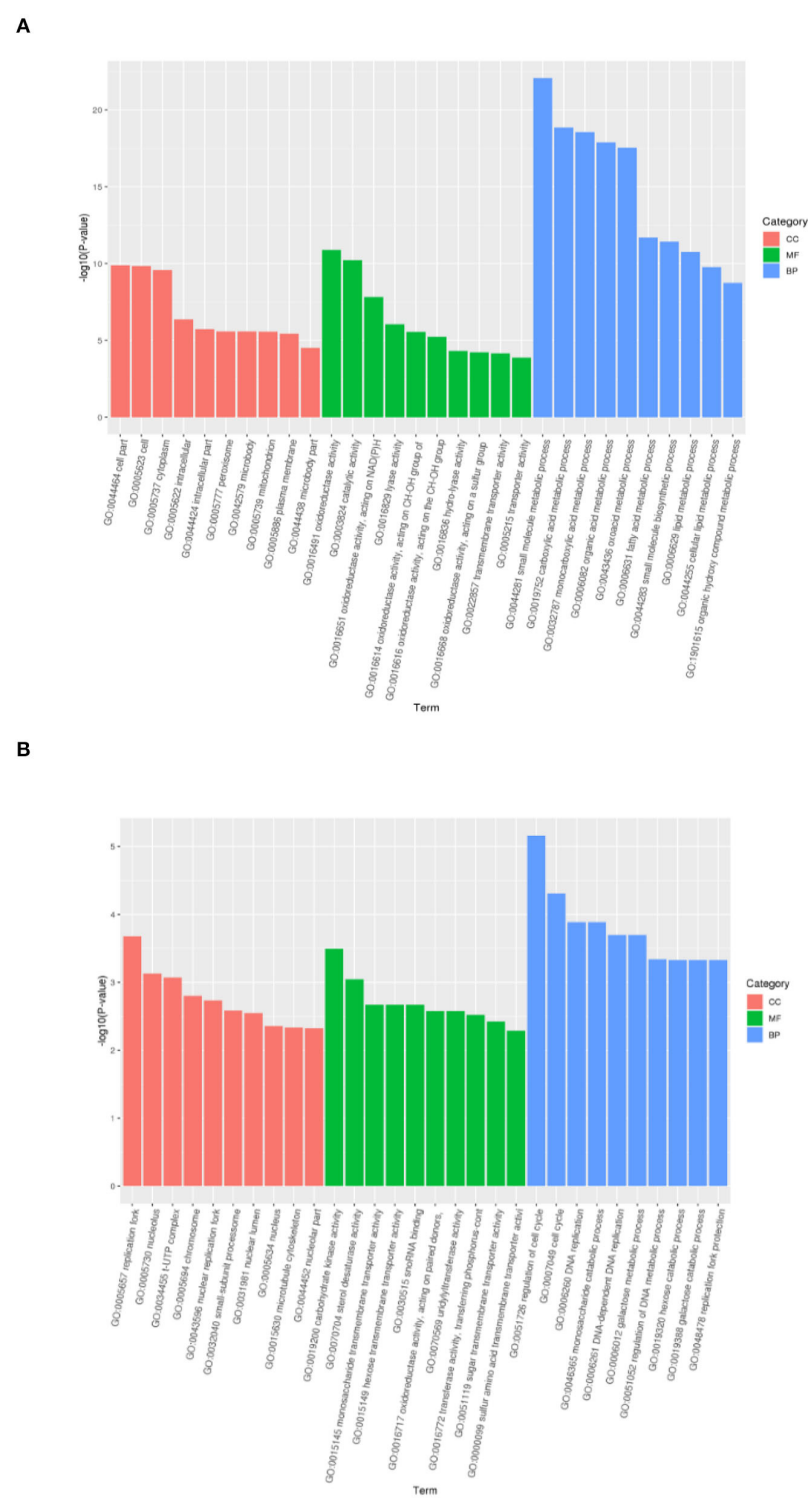
Significantly enriched GO terms of differentially expressed genes between the normal and VBNC cells **(A)** and between the VBNC and resuscitated cells **(B)**.

In order to determine the key molecular pathways responsible for the induction and recovery of VBNC *B. bruxellensis* cells, GO-term function enrichment analysis was conducted independently to annotate the functions and metabolic pathways of DEGs in each pairwise comparison. Significantly enriched GO terms were divided into three main categories: biological process (BP), cell component (CC), and molecular function (MF). Figure 4 depicts the top 10 significantly enriched GO terms of the biological pathways with the lowest *P*-value in each classification category. As for the VBNC vs. NOR comparison group, the significantly enriched CC was involved in the peroxisome activity, MF in the oxidoreductase activity, and BP in the amino sugar and nucleotide sugar metabolism. Similar results are obtained in the RE vs. VBNC comparison group.

**Figure 5:**
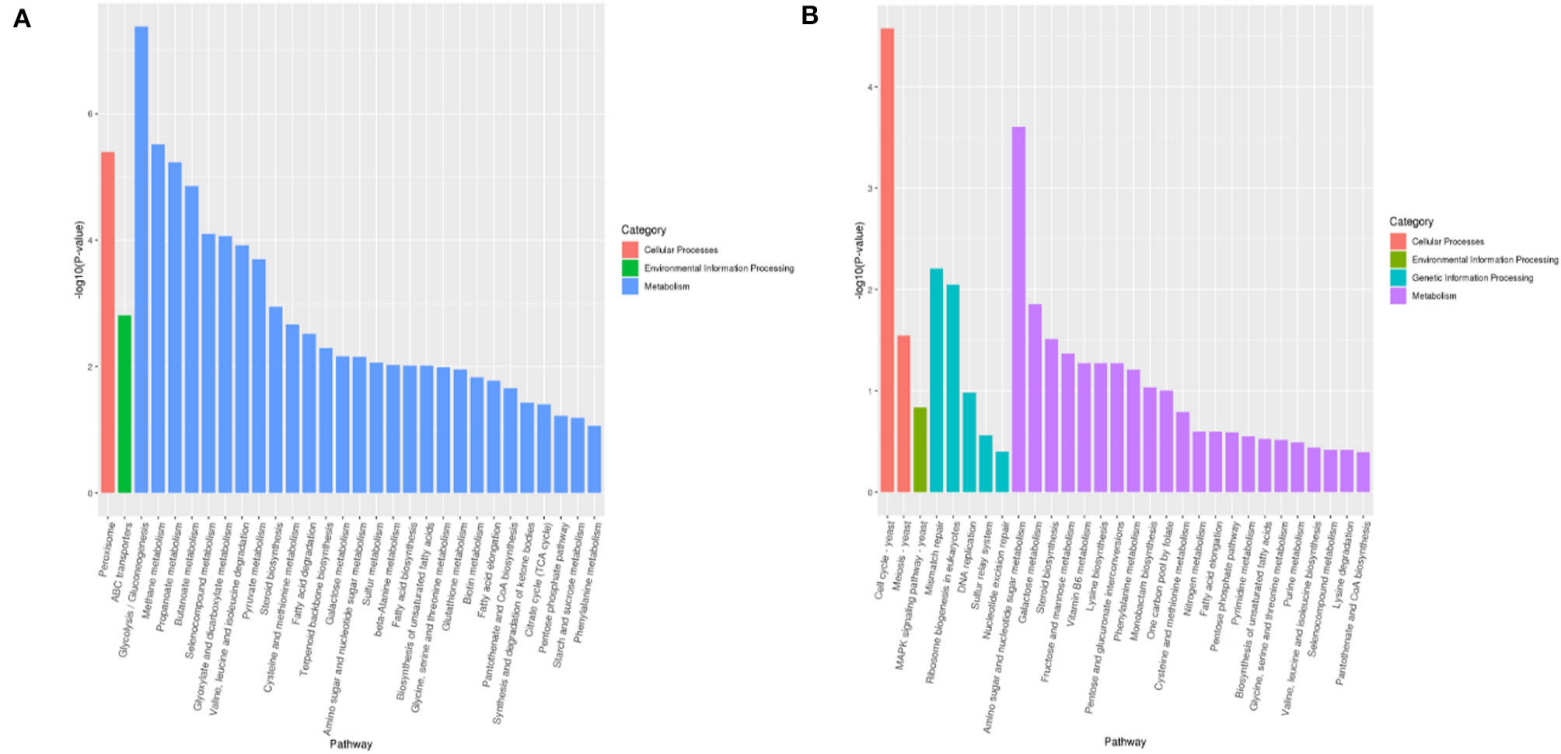
KEGG pathway enrichment of differentially expressed genes between the normal and VBNC cells **(A)** and between the VBNC and resuscitated cells **(B)**.

As shown in Figure 5, the top 30 significant pathways were obtained by KEGG enrichment analysis. As for the VBNC vs. NOR comparison group, significantly KEGG pathways included 28 metabolic pathways (methionine metabolism, glycolysis, methane, and cysteine, etc.), 1 peroxisome for cell engineering, and 1 for ABC transport in environmental information engineering. Furthermore, several molecular pathways, such as glycolysis, fatty acid, and methane metabolism, amino sugar and nucleotide sugar metabolism, nucleotide excision repair genetic information engineering, ABC transport, MAPK signaling pathway, and eukaryotic ribosome biogenesis could be responsible for the resuscitation of VBNC *B. bruxellensis*.

During the formation of VBNC *B. bruxellensis* cells, these up-regulated genes are most closely related to the ABC transporter, pyruvate metabolism, and peroxisome activity. It is well known that ABC transporters are important pathways for the import of essential nutrients and export of toxic molecules across the membrane in microorganisms, which can improve the stress resistance of microorganisms (Lai et al., 2009; Su et al., 2015; Liu et al., 2016). There are approximately 30 ABC transporters in yeast, and most of them are associated with the drug resistance such as pleiotropic drug resistance, PDR) transporters. *PXA1/2, PDR5*, and *SNQ2* located in this pathway were found to be significantly up-regulated. Simultaneously, enrichment of pyruvate metabolism *via* modulation of gene expression and enzymatic activity has been confirmed for microbial survival under acid stress or nutrient starvation (Liu et al., 2016). The peroxisome pathway plays a key role in reacting to oxidative stress. In the current study, *CAT, CuZnSOD*, and *DECR2* were significantly up-regulated in the peroxisome pathway. Indeed, several studies highlighted the entry into VBNC state of bacteria as the response to oxidative stresses (Lai et al., 2009; Su et al., 2015; Liu et al., 2016). This further explained why catalase could recover the VBNC *B. bruxellensis*. Other up-regulated genes in the category of homologous recombination, mismatch repair, and DNA replication also potentially revealed that VBNC *B. bruxellensis* was able to repair DNA damage caused by high concentrations of hop bitter acids. Meanwhile, the down-regulated genes were enriched for those encoding factors that function in carbohydrate metabolism, amino acid transport and metabolism, and oxidoreductase activity. This may be a useful strategy to reduce cell energy consumption and increase the function of survival systems when dealing with external adverse environment. Thus, *B. bruxellensis* cells could still retain their nutrient metabolism and protein synthesis when in the VBNC state.

Concerning the exit from the VBNC state, the main up-regulated pathways are closely related to carbohydrate metabolism, meiosis, nucleotide excision repair, basic transcription factors, and MAPK signaling pathways. Whereas the down-regulated pathways are mainly related to the organic acid metabolism and fatty acid metabolism. Our results indicated that the relevant pathways including carbohydrate metabolism, organic acid metabolism, and fatty acid metabolism might return to normal levels when the cells recovered from the VBNC state to the culturable state. On the other hand, the evidences of a general repression of genes involved in nutrient absorption and transportation, cell division, and DNA replication further confirm that the true cell recovery rather than a simple regrowth occurs. However, the key genes in the peroxisome pathway were not included in the KEGG database, thus their contribution to the formation of VBNC cells should be further verified by gene knockout methods.

Altogether, the *B. bruxellensis* cells produced extensive energy as a response to the hop bitter acids-stress, which destroyed the balance of energy distribution. In this event, the main regulatory genes contributing to VBNC entry and resuscitation included the genes related to carbohydrate and amino acid metabolism, indicating that the *B. bruxellensis* cells maintained weak protein synthesis and nutrient metabolism when they entered the VBNC state. At the same time, the peroxisome activity-associated genes were respectively up-regulated and down-regulated during the VBNC induction and resuscitation, suggesting that the *B. bruxellensis* increased cell tolerance against oxidative damage posed by hop bitter acids. Hence, the gene expression of the VBNC *B. bruxellensis* cells showed that the intense participant in stress response pathways and the decrease in key transport, metabolic, and enzymatic processes might be critically associated with their VBNC formation and recovery. Our transcriptome analysis on VBNC in eukaryotes shows the presence of frequent biological mechanisms shared with prokaryotes.

RNA-seq allows us to explore the molecular mechanisms of induction and recovery of various VBNC microorganisms. Recently, it was found that differential gene expression involved in the response to both oxidative stress and sulfite toxicity were obtained in the formation and resuscitation processes of sulfur dioxide-induced VBNC *B. bruxellensis* (Capozzi et al., 2016). Godoy et al. (2016) reported that the *p*-coumaric acid stress-response pathways in *B. bruxellensis* were involved in the expression of proton pump and efflux of toxic compounds. Asakura et al. (2007) noted that the differed expression of 29 DEGs in VBNC *Vibrio cholerae*, such as protein transport and binding, cellular processes, cell membrane pathways, and energy metabolism, was up-regulated or down-regulated by over 5 folds. Among the DGEs of *Vibrio parahaemolyticus* in the VBNC state induced by low temperature, the main up-regulated genes were connected to antioxidant, gluconeogenesis, and other pathways, and the down-regulated genes were involved in protein synthesis and ATP metabolism, etc. (Lai et al., 2009). Su et al. (2015) showed that DEGs in VBNC *Rhodococcus biphenylivorans* were associated with ATP metabolism, RNA polymerase and protein modification, and some enzymes (catalase and oxidoreductase). Our previous study demonstrated that the cold stress from beer production enhanced the gene expression of fatty acid synthesis and lipid metabolism of beer spoilage lactobacilli (Liu et al., 2016). In addition, Salma et al. (2013) found that the *SSU1* encoding the sulfite pump in *S. cerevisiae* yeast was only linked to the sulfur dioxide tolerance but not to the VBNC phenotype.

## Conclusions

Although the VBNC state has received widespread attention in bacteria, only few reports are focused on eukaryotic cells. In this work, the treatment with hop bitter acids (250 mg/L) for 2 h could induce complete entry of a beer-spoilage yeast *B. bruxellensis* into the VBNC state. These VBNC *B. bruxellensis* regained culturability after incubation on plates with 2,000 U/plate of catalase for 2 days. Transcriptome analysis was performed to identify the DRGs in the induction and recovery of the VBNC *B. bruxellensis*. GO function and KEGG pathway enrichment analysis were used to further determine the functional characterization of these DEGs and their related biological pathways. The genes associated with peroxisome activities, TCA cycle, ABC transporter, and organic acid metabolism were increased, whereas the carbohydrate and amino acid metabolism, DNA replication, and cell division were decreased in the VBNC *B. bruxellensis* cells. It was concluded that the promoted antioxidant cap, survival ability and declined cell division and metabolic activity in the *B. bruxellensis* cells might lead to the formation of the hop bitter acids-induced VBNC state. This research supplies a theoretical basis for microbial risk assessment in the brewing industry. However, it is noteworthy that the *Brettanomyces* has been described as a very diverse species both on genotypical and phenotypical levels. In order to reveal the molecular mechanisms that stimulate the formation and resuscitation of the VBNC state, further research is thus needed to collect and analyze more *Brettanomyces* strains isolated from contaminated beers.

## Data Availability Statement

The data presented in the study are deposited in the NCBI Sequence Read Archive (SRA) repository, accession number PRJNA833394.

## Author Contributions

YD and JZ contributed the experimental design. YH performed the experimental work. YD wrote the manuscript. YH, JZ, HY, and YD contributed to manuscript revision and read and approved the submitted version. All authors contributed to the article and approved the submitted version.

## Funding

This work was financially supported by the Open Research Fund of State Key Laboratory of Biological Fermentation Engineering of Beer (no. K201805).

## Conflict of Interest

YH and HY are employed by Tsingtao Brewery Co. Ltd. The remaining authors declare that the research was conducted in the absence of any commercial or financial relationships that could be construed as a potential conflict of interest.

## Publisher's Note

All claims expressed in this article are solely those of the authors and do not necessarily represent those of their affiliated organizations, or those of the publisher, the editors and the reviewers. Any product that may be evaluated in this article, or claim that may be made by its manufacturer, is not guaranteed or endorsed by the publisher.
